# Extra-Cavitary Primary Effusion Lymphoma in a Patient Co-infected With HIV-1 and HIV-2: A Case Review

**DOI:** 10.7759/cureus.75039

**Published:** 2024-12-03

**Authors:** Maria João Miguel, Tomás Robalo Nunes, André Gomes, Susana Boavida, Nuno Marques

**Affiliations:** 1 Infectious Diseases, Hospital Garcia de Orta, Lisbon, PRT

**Keywords:** aids related cancers, dual infection with hiv-1 and hiv-2, hiv, human herpes virus 8, non-hodgkin lymphoma, primary effusion lymphoma

## Abstract

Extra-cavitary primary effusion lymphoma (PEL), often associated with human herpes virus 8 (HHV8) infection, represents a rare and aggressive form of non-Hodgkin lymphoma, which is predominantly found in individuals with severe immunosuppression. As an acquired immunodeficiency syndrome (AIDS)-associated lymphoma, PEL typically manifests in the context of advanced human immunodeficiency virus (HIV) infection, requiring tailored therapeutic approaches to manage both the lymphoma and underlying immunodeficiency.

A 53-year-old male patient from Cape Verde presented with a three-day history of fever, night sweats, right iliac fossa pain, hematochezia, and an unintentional weight loss of five kilograms over the previous two months. A laboratory study revealed a previously undiagnosed co-infection with HIV-1 and HIV-2, with a CD4+ T-cell count of 63/μL. The abdominal-pelvic computed tomography (CT) scan revealed hepatosplenomegaly with hypodense nodular lesions and prominent lymph nodes in the celiac-mesenteric, axillary, and cervical regions. Lung imaging showed non-specific nodules. An extensive investigation for opportunistic infections was conducted, with a bronchoalveolar lavage culture test positive for *Mycobacterium xenopi *and a colon biopsy indicating Cytomegalovirus colitis. Further histological examination from a gastric biopsy revealed the diagnosis of the solid variant of PEL. The patient initiated treatment for opportunistic infections followed by antiretroviral therapy. However, he experienced multiple complications and due to his deteriorating condition, chemotherapy was not initiated and he ultimately died.

This rare clinical case of lymphoma in a patient co-infected with HIV-1 and HIV-2, the first of its kind to be reported, to the authors' knowledge, underscores the diagnostic and therapeutic challenges associated with this condition.

## Introduction

The life expectancy of people living with human immunodeficiency virus (PLWH) under antiretroviral therapy has increased over time and is now only a few years shorter than that of the general population [1]. Consequently, deaths related to acquired immunodeficiency syndrome (AIDS) have been gradually decreasing [2]. However, mortality due to both AIDS-related and non-related cancers remains high [3].

AIDS-associated non-Hodgkin (NH) lymphomas represent more than 50% of all AIDS-defining cancers, including systemic lymphoma, primary central nervous system lymphoma, and primary effusion (or body cavity) lymphoma (PEL) [4]. The World Health Organization (WHO) classifies PEL as a type of large B-cell lymphoma, [5] accounting for approximately 4% of AIDS-associated lymphomas [4]. PEL can manifest in two distinct forms: the classical form, characterized by significant neoplastic effusions in the body cavities without detectable tumor masses, and the extra-cavitary form, where detectable masses may occur in lymph nodes, the gastrointestinal tract, central nervous system, and skin [4,5].

Although its pathogenesis is not completely understood, PEL is associated with infection by human herpes virus 8 (HHV8)/Kaposi sarcoma (KS)-associated herpes virus (KSHV) [5]. Co-infection with Epstein-Barr virus (EBV) is also present in 60-90% of the cases [4]. PEL has a poor prognosis [6], with an overall survival at one year of approximately 30% [5].

To the authors' knowledge, extra-cavitary PEL in a co-infected patient with HIV-1 and HIV-2 has not been described before.

## Case presentation

A 53-year-old male patient, originally from Cape Verde and residing in Portugal for the past 14 years, presented to the Emergency Department in 2017 with a three-day history of fever (peaking at 38.8°C), night sweats, right iliac fossa pain, and hematochezia (two to three bowel movements per day). He reported an unintentional weight loss of five kilograms over the previous two months but denied other gastrointestinal, respiratory, or genitourinary symptoms. His past medical history was unremarkable, and he was not on any regular medications.

On examination, the patient appeared underweight and febrile, with dehydrated skin. Abdominal palpation revealed tenderness in the right iliac fossa, and several tender, centimeter-sized lymph nodes were noted in the cervical and inguinal regions. Cardiopulmonary auscultation was normal.

Laboratory tests indicated anemia (hemoglobin 7.9 g/dL; reference range: 11.5 - 18.0 g/dL) and thrombocytopenia (97 x 10^9/L; reference range: 130 - 400 10^9 /L). A fourth-generation HIV test was positive, and a Western blot confirmed co-infection with HIV-1 and HIV-2, with viral loads of 512,348 copies/mL and 179 copies/mL, respectively. Notably, the patient was unaware of his HIV status. Immunologically, he had a CD4+ T-cell count of 63/μL (2.1%; reference range: 500 - 1.500 cells/μL (percentage: 40.00 - 59.00%). The HIV-1 resistance test showed no resistance to protease inhibitors or reverse transcriptase inhibitors. The serum cryptococcal antigen test was negative.

**Figure 1:**
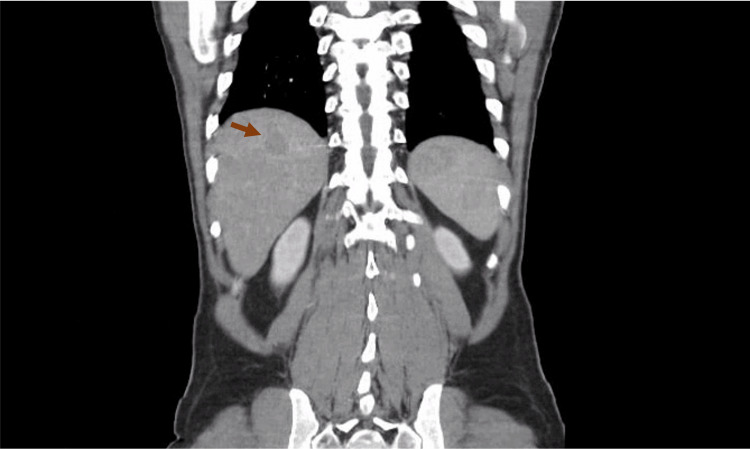
Abdominal CT Scan Hepatosplenomegaly with hypodense nodular lesions (arrow) in the liver and prominent lymph nodes in the celiac-mesenteric region.

A full-body computed tomography (CT) scan revealed hepatosplenomegaly with four hypodense nodular lesions in the liver (Figure 1) and prominent lymph nodes in the celiac-mesenteric, axillary, and cervical regions. Lung imaging showed non-specific nodules in the left upper lobe and middle lobe.

Bronchoscopy revealed acid-fast bacilli in bronchoalveolar lavage (BAL); however, the sample was insufficient for molecular analysis, prompting empiric antimycobacterial treatment with isoniazid, rifampin, pyrazinamide, and ethambutol. Despite this, the patient’s fever persisted, and BAL cultures remained negative. A repeat bronchoscopy yielded negative results for Mycobacterium tuberculosis, leading to the cessation of antimycobacterial therapy. Three months later, the results of the BAL culture test returned positive for non-tuberculous mycobacteria. Molecular analysis identified this as Mycobacterium xenopi. As a result, the patient was started on a treatment regimen that included isoniazid, rifampicin, ethambutol, and clarithromycin.

In parallel, alternative diagnoses were investigated. A liver biopsy indicated chronic hepatitis with granulomas, but immunophenotyping did not reveal pathological lymphoid populations. Bone marrow examination revealed 6% lymphocytes with non-specific morphological abnormalities, likely reactive to infection, and immunophenotyping analysis confirmed the absence of pathological lymphoid populations. A cervical lymph node biopsy yielded similar results.

Due to recurrent hematochezia and intermittent diarrhea, the patient underwent a colonoscopy, revealing hyperemic and eroded mucosa in the terminal ileum, cecum, transverse colon, and sigmoid colon. Colon biopsy results indicated Cytomegalovirus (CMV) colitis, displaying characteristic cytoplasmic and nuclear inclusions, with positive immunohistochemistry for CMV.

**Figure 2:**
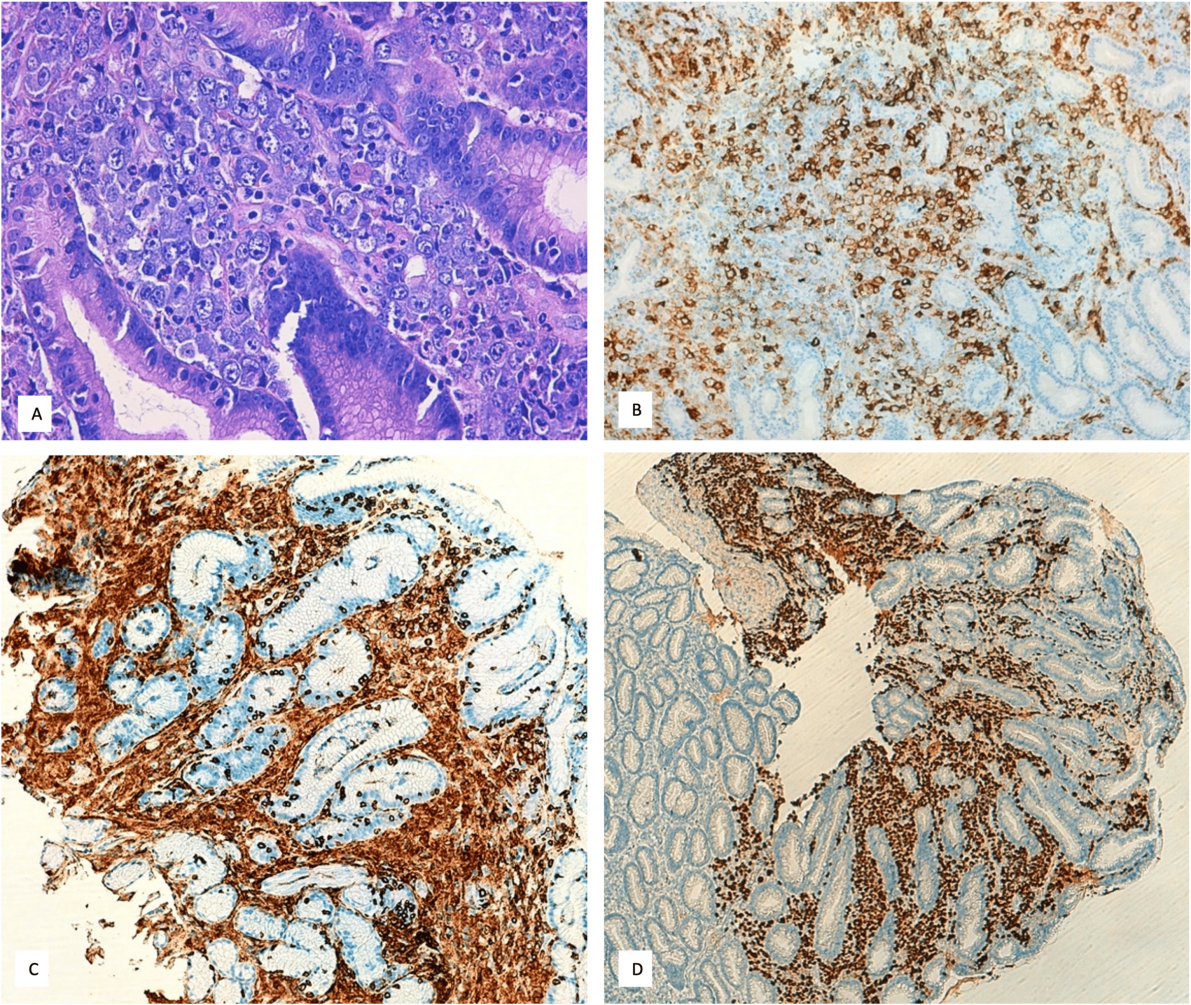
Histology Histological section of a gastric biopsy stained with hematoxylin and eosin (H&E) showing clusters of abnormal lymphoid cells infiltrating the gastric mucosa (A). Immunohistochemical results: the tumor cells in the gastric biopsy show partial expression of CD30 (B); tumor cells positive for CD45 (C); nuclei of tumor cells positive for HHV-8 (D). HHV-8: Human herpes virus 8

An upper gastrointestinal endoscopy showed whitish-speckled mucosa in the esophagus (suggestive of candidiasis) and areas of marked hyperemia in the stomach. Gastric biopsy results confirmed a diagnosis of high-grade lymphoma, consistent with the solid variant of PEL. The infiltrate consisted of pleomorphic cells, with the following immunophenotype: CD45+, CD30+ (partial), CD138+, HHV8+, EBV (CISH)+, CD4+ (focal), CD3-, CD5-, CD2-, CD8-, CD56-, ALK-, CD15-, CD20-, PAX5-, CKs-, S100-, and MPO- (Figure 2).

The patient began treatment for esophageal candidiasis and CMV colitis and trimethoprim-sulfamethoxazole prophylaxis, followed by initiation of a triple antiretroviral therapy regimen based on an integrase inhibitor, for a patient without resistance-associated mutations, with raltegravir, tenofovir disoproxyl fumarate, and emtricitabine. 

Unfortunately, he experienced multiple complications, including methicillin-sensitive Staphylococcus aureus bacteremia linked to a peripheral venous catheter, which progressed to endocarditis. Due to his deteriorating condition, chemotherapy was not initiated. The patient ultimately died due to heart failure secondary to Staphylococcus aureus endocarditis, likely compounded by progression of the lymphoma and uncontrolled HIV infection.

## Discussion

AIDS-defining illnesses continue to be the leading cause of death among PLWH. However, significant progress in antiretroviral therapy has led to a notable reduction in the proportion of AIDS-related mortality. Consequently, there has been an increase in deaths due to cardiovascular diseases and non-AIDS-defining cancers [2]. Currently, non-AIDS-related cancers are more frequently diagnosed than those related to AIDS [7].

In this case report, the patient likely succumbed to complications associated with AIDS, specifically due to progression of AIDS-defining cancer which contrasts with the overall trend in the antiretroviral era. A critical factor in this scenario was the late diagnosis of HIV, defined as presenting for care with a CD4 count below 350 cells/mL or with an AIDS-defining event, irrespective of CD4 count, [8] excluding acute infections [9]. In Europe, the proportion of late diagnoses has decreased over the past decade; however, it remained significant at 48.9% in 2022 [10]. In Portugal, this figure was even higher, with 57.2% of new diagnoses classified as late presentations, and 34.2% had CD4 counts below 200 cells/μL. Among patients of African descent in Portugal, such as our patient from Cape Verde, the late presentation rate was even more concerning, at 63.2% in 2022 [11].

Particularly our patient presented with co-infection with HIV-1 and HIV-2, diagnosed following the most recent HIV-2 guidelines [12].

HIV-2 infection is primarily limited to West Africa and countries with historical, social, and economic ties to this region, like Portugal and its former colonies, [13] which applies to our patient. Compared to HIV-1, HIV-2 progresses more slowly, [12] resulting in longer median survival times and a longer time for the development of AIDS [14]. Furthermore, individuals infected with HIV-2 typically develop clinical AIDS at higher mean CD4 percentages than those infected with HIV-1 [14]. In addition, HIV dual infection may not be a static condition, and there are theories that suggest that HIV-1 effectively overgrows HIV-2 in the dually exposed host individual [15].

Our patient was diagnosed with four AIDS-defining illnesses: the more common CMV colitis and esophageal candidiasis, the non-tuberculous mycobacteria infection, and the rare PEL. As far as the authors know, this is the first reported case of extra-cavitary PEL diagnosed in an HIV-1 and 2 co-infected patient.

PEL, associated with HHV-8, is a rare form of NH lymphoma typically diagnosed in immunocompromised patients, particularly those with HIV. While diffuse large B-cell lymphoma and Burkitt lymphoma are the most common NH lymphomas in the AIDS population, PEL accounts for approximately 4% of HIV-associated NH lymphomas [6].

The classical presentation of PEL often involves malignant effusions in the pleural, peritoneal, or pericardial cavities [4-6]. In 2004, Chadburn et al. proposed that the rare extra-cavitary or solid variant of KSHV-positive solid lymphoma should be considered part of the same clinical spectrum as PEL [16]. Morphologically, immunophenotypically, and molecularly, classical and extra-cavitary PELs share many characteristics [16]. However, this classification has been debated, leading to the suggestion of a consensus diagnostic term: "KSHV-associated large B-cell lymphoma (KSHV-LBL)" [17].

Typically, patients diagnosed with PEL are male and present with advanced HIV infection, often with CD4 counts below 200 cells/μL [6,16]. A retrospective study conducted in France from 1996 to 2013 found that a significant proportion of PEL patients had experienced severe immune deficiency, with 65% having a prior history of AIDS-defining conditions, and 25% still exhibiting CD4 counts below 200 cells/μL at PEL diagnosis [6]. Our patient aligned with this data, presenting with advanced immunosuppression (CD4 count of 63 cells/μL) and multiple concomitant opportunistic infections.

In this case, the patient exhibited non-specific symptoms, including weight loss, fever, and night sweats, which are common in cases of advanced HIV infection, complicating the clinical diagnosis of PEL. Other reports also pointed to systemic B symptoms as the most prominent [6].

The described patient had documented PEL detection in the gastrointestinal tract. When this clinical entity was first described, the primary site of infection was identified as the lymph nodes and the chest wall [16]. In a series of 17 patients with extra-cavitary PEL, the most frequently involved organs included lymph nodes (35% of cases), but also the gastrointestinal tract, spleen, central nervous system, bone marrow, liver, and skin [6].

Pathological diagnosis of PEL is complex, requiring the detection of HHV-8 in neoplastic cells via immunohistochemistry or quantitative DNA amplification. While co-infection with EBV is common, it is not essential for diagnosis [6] with some cases reported as EBV-negative [16,18]. Research suggests that EBV status does not influence survival outcomes, as HHV-8 is the primary driver of the disease, even in cases of co-infection [6].

Due to its low incidence, no randomized clinical trials have established standard treatment protocols for PEL [6]. Proposed treatments include CHOP-like regimens, with or without high-dose methotrexate, although the benefit of adding methotrexate remains debated [6]. Additionally, the role of antiretroviral therapy is crucial in managing this condition, as it controls HIV infection and improves CD4 counts [16]. Notably, the prognosis for PEL patients improved significantly following the widespread introduction of antiretroviral treatment [5]. The absence of such therapy before diagnosis has been identified as an independent risk factor for increased mortality [18]. In our case, triple antiretroviral therapy based on an integrase inhibitor was started, which is active against both HIV-1 and 2 [12].

A recent case report highlighted an HIV-infected patient with PEL who underwent an autologous bone marrow transplant, achieving complete and sustained remission eight years post-diagnosis [19].

The prognosis for PEL remains poor, with median survival rates as low as 6.2 months [18]. While some studies suggest no significant difference in survival between classical and extranodal forms of PEL, [6] others propose a potentially better prognosis for the extra-cavitary variant [16]. Unfortunately, in our patient's case, the diagnosis of the extranodal form coincided with the advanced stage of HIV infection. The progression of the disease, coupled with immunosuppression and hospitalization complications, such as nosocomial infections and the outcomes associated with severe infectious endocarditis, ultimately contributed to the patient's death.

## Conclusions

To the authors' knowledge, this is the first reported case of extra-cavitary PEL in a patient co-infected with both HIV-1 and HIV-2. The limited literature on HIV-1 and HIV-2 co-infection presents significant challenges in managing these patients and their associated opportunistic infections. Further research is crucial to optimize treatment strategies and improve patient outcomes.
